# Towards forming a socio-ecological action model for urban open spaces’ design in New Cairo, Egypt

**DOI:** 10.1186/s44147-021-00005-z

**Published:** 2021-08-04

**Authors:** Dalia M. Rasmi, Mohamed A. Zayed, Khaled M. Dewidar, Hisham S. Gabr

**Affiliations:** 1 Department of Architecture, Faculty of Engineering, Cairo University, Giza, Egypt; 2 Department of Architecture, Faculty of Engineering, The British University in Egypt, Cairo, Egypt

**Keywords:** Urban open spaces, Urban design, Urban attributes, S.E.A.M’s matrix, Physical elements, Social and cultural aspects, Ecological and natural variables, Thematic analysis

## Abstract

Under the supervision of UN-Habitat, the Egyptian General Organization of Physical Planning started its first phase of “Promoting Better Quality and More Manageable Public Spaces Project, 2021” that targets enhancement and development of open spaces quality in New Cairo, Egypt. This project is functioning under three main objectives: (1) recognize the most occupied urban open spaces in New Cairo, (2) identify the required community needs in these urban open spaces, and (3) evaluate quality and suitability of these open spaces for public usage. In this paper, we are attempting to achieve the 2nd objective addressed previously by laying hands on hidden correlations among socio-ecological community needs. This is achieved in two phases; the first phase is mainly concerned with adapting thematic analytical method to tackle multiple correlations while reviewing literature, while the second phase is focusing on conducting a pilot study survey in East Academy district to validate the previously concluded socio-ecological correlations. Also findings indicate that East-Academy’s open spaces have strong correlations with multiple socio-ecological attributes and that ten urban qualities showed the highest positive measures. These correlations, in the future, can be used to establish an intervention action model.

## Introduction

We may claim that what defines social ecology as social is that almost all of our present ecological crises are a reflection of deep social problems. As stated by Janlin in 2012, “to separate ecological from social problems would be to grossly misconstrue the sources of the growing environmental crisis” [1]. Therefore, the way human-beings deal with each other as social beings is crucial to address our current ecological crisis. Reaching such change requires transformation of our mentality from domination to complementarity, in which our role will be shifted to being supportive and appreciative of the non-human life’s needs. This concept was initially presented in 1993 during the first public statement to advance the idea of social ecology, this statement claimed that “The cast of mind that today organizes differences among human and other life-forms along hierarchical lines of ‘supremacy’ or ‘inferiority’ will give way to an outlook that deals with diversity in an ecological manner, according to an ethics of complementarity” [2].

In 2021 due to the COVID-19 pandemic, a global insight towards importance, quality, and suitability of urban open spaces is being magnified [3]. This paper comes aligned with this governmental decision to develop New Cairo urban open spaces as a pilot study for a community with better quality of life [4]. However, investing in green infra-structure together with energy efficient strategies was not of a concern. This derived a demand and need for transdisciplinary action strategies or guidelines to help in designing and assessing new urban communities. How can we design a space that is both socially accepted and ecologically oriented is a key question that this paper is trying to answer by filling the gap between literature and practice; this will help in showing broad lines for a pathway towards socio-ecological design. Therefore, this paper aims to achieve what was previously explained above by analysing literature, showing different themes of relations between socio-ecological variables and drawing action strategies that can be used as a framework for an eco-city model in Egypt, such as renewable energy, permaculture, environmental design, eco-waste management, green transportation, and green cities.

### Social ecology

The sociology field has an over lapping concerns with related disciplines; some scholars have begun to doubt whether sociology does have a clear focus of its own or not [5]. Thus, sociology focuses on social psychology, social stratification, the new sociology, demography, and social problems at the expense of other important approaches such as ecology, psychology, and social spatial patterns.

Sociology is so complex and diversified concept that cannot be interpreted from a single approach. What is needed is a multidimensional approach with multiple perspectives to highlight the relationship between different concepts. Moreover, researchers cannot discuss communities’ life without taking into consideration the following questions: How did these communities get to be the way they are? And what are the existing forces likely to produce change? Any efforts to describe the current social realities are difficult without mentioning the social change. By time, social patterns are the urban representation of social change; when observed it can be analysed by tracing its “Physical Traces” [6].

### The ecological perspective

Natural environment has been neglected as a topic of concern from ecologists for years. Sociologists have been also criticized for failing to adequately deal with physical environment and social factors interrelationships [7]. Meanwhile, ecology is concerned with the processes and forms of people’s adjustments to their physical environment. More specifically, the study of territorially based on spatial systems created by human efforts has come to be known as “ecology” [8]. Otis has gone further in viewing community as an ecological system; his concept identifies the major four classes of eco-system elements as follows: population, environment, technology, and social organization.

### The cultural perspective

Cultural perspective components include values, norms, sanctions, and symbols. The point being made here is that both material (physical) and nonmaterial (non-physical) cultural aspects are integral components of the physical structure and social life of all human communities [9].

### Environmental psychology

Environmental psychology is the science that examines the relationship between humans and their environment using tools, such as users’ needs assessments known as PDR to evaluate requirements prior to design. Environmental psychologists help to understand the differences between temporary and long-term needs. This includes a multidisciplinary approach to understand human behavioural response and motive as well. Theories in the human environment relationship aid the process of understanding the users’ needs before a design is created. They include integration, stimulation, control, and behaviour setting.

Generally, these theories explain the stimulation of human behaviour relationship such as the arousal perspective, environmental load, and adaptation. These theories of environmental properties are pleasure-arousal-dominance hypothesis [10], Kaplan and Kaplan [11] preference framework [12], and Lynch’s elements [13] of legibility [14].

### Environmental psychology models, perspectives, and implementation

Theories, models, and perspectives in environmental psychology are presented in Table 1.

**Table 1:** Theories, models, and perspectives in environmental psychology [15]; edited by Dalia M. Rasmi (author) 2021

Theories and models		Theorist	Major premise
**01**	**Social learning theory**	Albert Bandura	Determines that we learn by first observing others and reproducing their actions.
**02**	**Integration theory**	Anne Treisman and Garry Gelade	Elements of the environment work in harmony to facilitate a particular behaviour.
**03**	**Control theory**	Walter Reckless	Group of theories that address behavioural constraints and a person’s perceived control over his or her actions and behaviours.
**04**	**Behaviour setting theory**	Roger Barker	Public places or settings evoke certain patterns of behaviour.
**05**	**Stimulation theory**	Nick Bostrom	Environment is a source of sensory information (stimuli) that leads to arousal.
**06**	**Lens model**	Kennth Hammond	Stimuli from the environment become focused through our perceptions.
**07**	**Affordances**	James Gibson	The world is composed of substances, surfaces, and textures, the arrangement of which provides recognizable function of environmental features.
**08**	**Collative prosperities**	Daniel Berlyne	We respond to aesthetics based on their collative properties.
**09**	**Pleasure-arousal-dominance hypothesis**	Mehrabian and Russell	Three primary emotional responses are translated to positive feelings, excitement, and control over the setting with pleasure and arousal as the two main axes.
**10**	**Preference model**	Lichtenstein and Slovic	People prefer engaging scenes to boring scenes.
**11**	**Elements of legibility**	Kevin Lynch	Five predominating qualities enhance its legibility to the average person.

### Environmental psychology concepts and its implementations

Environmental psychology concepts are presented in Table 2.

**Table 2:** Environmental psychology concepts 13; Dak Kopec in 2012

	Key concepts	Relevance for design
**01**	**Reciprocal determinism, modelling**	Encourages an understanding of established societal norms
**02**	**Global environment, instigators, goal objects, supports and constraints, directors**	Offers a holistic approach to design
**03**	**Psychological reactance**	Suggests that design elements lead to perceptions of control
**04**	**Operant conditioning, interactional theory**	Emphasizes that design is an important component of a setting that contributes to certain behaviours
**05**	**Threshold, arousal, environmental load, overload, adaptation level**	Hold that design styles can lead to over-or under-stimulation
**06**	**Directed attention, attentional deficit, effortless attention, restorative experiences**	Include views of green spaces for effortless attention within environments demanding much directed attention
**07**	**Distal and proximal cues leading to cue validity and cue utility**	Emphasizes that perceptual relationship between design and the human observer
**08**	**Environmental layout, contextual cues direct perception**	Highlights perceptual influences of design styles and probable dual uses of designs
**09**	**Novelty, incongruity, complexity, surprise, hedonic tone, uncertainty-arousal**	Claims that the joint nature of design elements merge to develop one overall impression
**11**	**Pleasure, arousal**	Offers a method to evaluate environmental designs
**12**	**Coherence, legibility, complexity, mystery**	Offers method for designing engaging environments
**13**	**Paths, edges, districts, nodes, landmarks**	Offers a method to enhance an environment’s legibility

### Action models

#### Place making model (PMM), Project for Public Spaces 2013

**Fig. 1:**
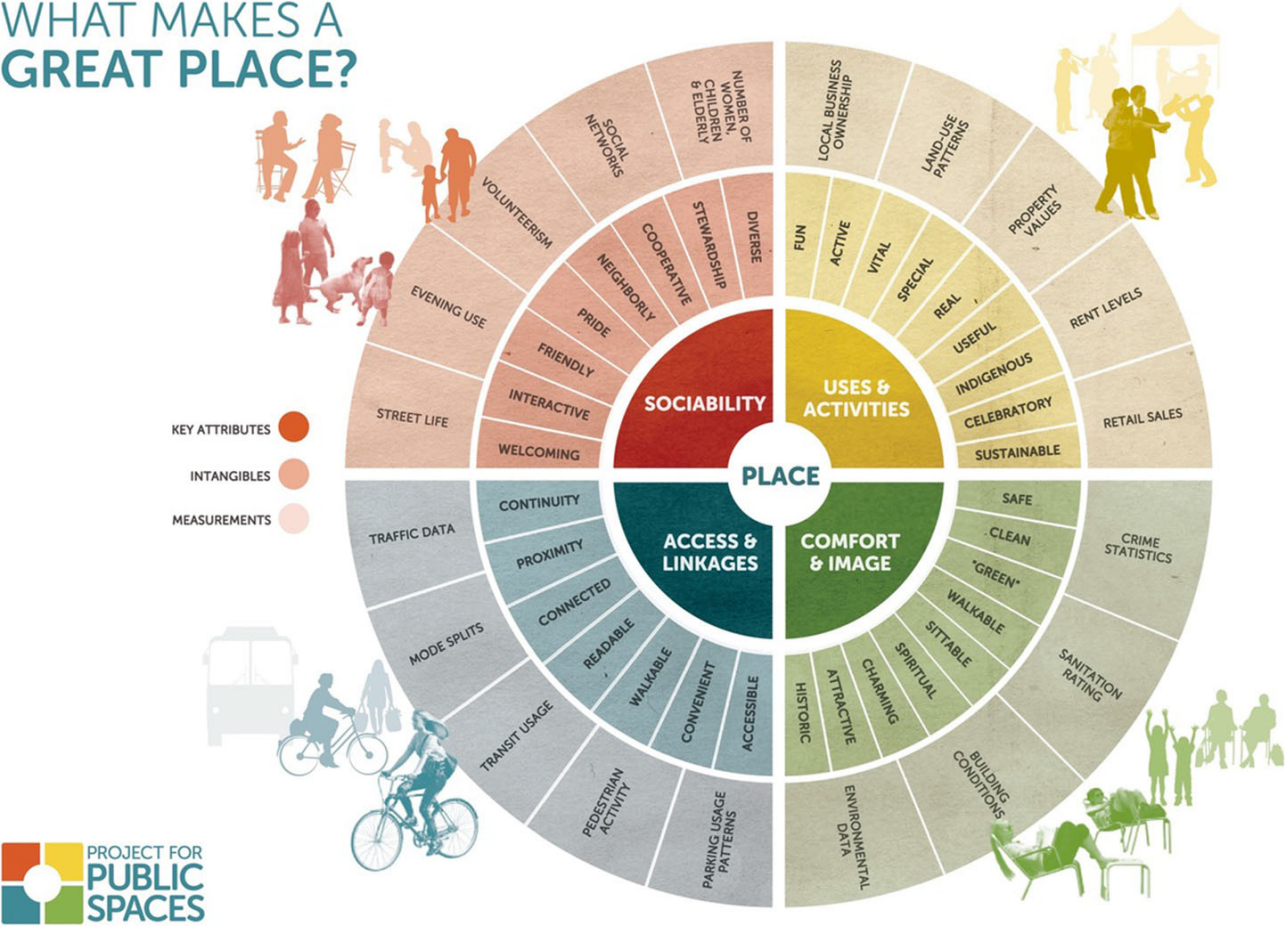
Place making model by project for public spaces in 2013–2019 [17]

Project for public spaces (PPS) organization in 2013 has found that successful public spaces usually share four main qualities: being accessible, engaging people with physical and mental activities are comfortable, and have a positive image, in addition to being perceived as sociable spaces [16]. After evaluating thousands of open public spaces around the world, PPS developed a model that is called “The Place Making Model” as shown in Fig. 1. This model plays a role as a diagnostic tool for any space whether good or bad [17]. In Fig. 1, a specific open space such as a street, plaza, or play-ground can be evaluated according to four main criteria that are displayed in the inside ring. Outside of this ring are a number of qualitative aspects by which to judge a space accordingly. Finally, the outer ring shows the quantitative aspects that can be measured by statistical research.

### Matrix of S.E.A.M’s variables

The matrix of social ecology evolved from multiple sources of knowledge that differ from books to theories. A comprehensive reading and a thematic analysis took place to establish the base for S.E.A.M’s variables formation. In an ascending state, according to date of publication, nine main sources are used to extract these variables: The book of Santayana “The Sense of Beauty” published in 1955 [18], the mental map elements by Kevin Lynch in 1960 [13], the “Pattern Language” by Christopher Alexander in 1977 [19], “Human Aspects of Urban Form” by Amos Rapoport in 1977 [20], “Creating Defensible Spaces” by Oscar Newman in 1973 [21], the eco master planning (the four infra-structures) by Ken Yeang in 2009 [22], the variables of “Ecological Urbanism” by Mohsen Mostafavi in 2016 [23], variables of “Environmental Psychology” by Dak Kopec in 2012 [15], and lastly Helen Woolley’s variables of “Urban Open Spaces” in 2013 [24] (Tables 3, 4, and 5). Moreover, a comparative analysis between all the previously mentioned variables and “The Place Making Model” from project for public spaces formed in 2013 is explored to achieve the final matrix of S.E.A.M’s variables [15].

**Table 3:** S.E.A.M’s matrix of physical and spatial elements

Code	Attributes	Source
**Physical and spatial elements**		
**01**	**Plants (flora and fauna)**	Helen Woolley, 2013
**02**	**Land marks and attraction points**	Kevin Lynch, 1960
**03**	**Enclosure and openness**	Oscar Newman, 1973–1996
**04**	**Historical elements**	Kevin Lynch, 1960
**05**	**Space furniture**	Dak Kopec, 2012
**06**	**Accessibility**	Project for Public Spaces, 2013
**07**	**Continuity**	Project for Public Spaces, 2013
**08**	**Proximity**	Project for Public Spaces, 2013
**09**	**Connectivity and movement**	Christopher Alexander, 1977
**10**	**Way finding and navigation**	Kevin Lynch, 1960
**11**	**Walkability**	Project for Public Spaces, 2013
**12**	**Mixed uses and services**	Christopher Alexander, 1977

**Table 4:** S.E.A.M’s matrix of social and cultural aspects

Social and cultural aspects		
**13**	**Safety and security**	Oscar Newman, 1973–1996
**14**	**Visibility and surveillance**	Oscar Newman, 1973–1996
**15**	**Crime prevention**	Oscar Newman, 1973–1996
**16**	**Comfort level**	Dak Kopec, 2012
**17**	**Activities (active\passive)**	Helen Woolley, 2013
**18**	**Motivation**	Project for Public Spaces, 2013
**19**	**Heritage values**	Santyana, 1955
**20**	**Memories**	Dak Kopec, 2012
**21**	**Space attachment**	Amos Rapoport, 1977
**22**	**Sense of beauty**	Santyana, 1955
**23**	**Entertainment and pleasure**	Dak Kopec, 2012
**24**	**Group membership and community ties**	Amos Rapoport, 1977
**25**	**Stewardship\leadership**	Amos Rapoport, 1977
**26**	**Co-operation**	Amos Rapoport, 1977
**27**	**Participation and Engagement**	Amos Rapoport, 1977
**28**	**Interaction with human\nature**	Dak Kopec, 2012
**29**	**Social ties and friendship**	Amos Rapoport, 1977
**30**	**Sense of pride**	Amos Rapoport, 1977
**31**	**Diversity and variation**	Project for Public Spaces, 2013
**32**	**Social cohesion**	Amos Rapoport, 1977

**Table 5:** S.E.A.M’s matrix of ecological and natural variables

Ecological and natural variables		
**33**	**Green infra-structure**	Ken-Yeang, 2009
**34**	**Blue infra-structure**	Ken-Yeang, 2009
**35**	**Waste management**	Mohsen Mostafavi, 2016
**36**	**Recycled materials**	Mohsen Mostafavi, 2016
**37**	**Pedestrian paths**	Kevin Lynch, 1960
**38**	**Space maintenance**	Newman, 1972; Kelling, 1982

“The Place Making Model” (31) parameters in Table 6 were cross examined with many variables concluded from literature to formulate the final representation of S.E.A.M’s matrix of 38 variables in Tables 3, 4, and 5 that will be latterly used to trace correlations. In Table 6, the first column represents PPS four main sectors: image and comfort, uses and activities, sociability, and access and linkages. Moreover, the second column demonstrates the list of PPS variables associated with each category. Furthermore, the third and fourth columns relate S.E.A.M’s attributes by code and name to each PPS attribute. However, the previously mentioned S.E.A.M attributes are originally branched from three main categories as mentioned in Tables 3, 4, and 5.

**Table 6:** Cross examination between PPS and S.E.A.M’s attributes

No.	“PPS” main categories	No.	“PPS’s” attributes	S.E.A.M code	“S.E.A.M’s” attributes
1	**Image and comfort**	1	**Safety**	13	Safety and security
				14	Visibility and surveillance
				15	Crime prevention
		2	**Greenery and water features**	33	Green infra-structure
				34	Blue infra-structure
				01	Plants
		3	**Cleanliness**	35	Waste management
				36	Recycled materials
		4	**Attractiveness**	02	Landmarks and attraction
		5	**Relaxation**	16	Comfort level
				17	Activities (passive)
		6	**Welcome-ness**	03	Enclosure and openness
		7	**Motivation**	18	Motivation
		8	**Historical**	04	Historical elements
				19	Heritage values
		9	**Spirituality**	20	Memories
				21	Space attachment
				22	Sense of beauty
		10	**Seat-ability**	05	Space furniture
2	**Access and linkages**	11	**Accessibility**	06	Accessibility
		12	**Continuity**	07	Continuity
		13	**Proximity**	08	Proximity
		14	**Connectivity**	09	Connectivity and movement
		15	**Readability**	10	Way finding and navigation
		16	**Walkability**	11	Walkability
				37	Pedestrian paths
		17	**Convenience**	00	Satisfaction level*
3	**Activities and uses**	18	**Fun**	23	Entertainment and pleasure
		19	**Passive**	17	Passive activities
		20	**Special**	20	Memories
				21	Space attachment
		21	**Useful**	12	Mixed uses and services
		22	**Celebratory**	17	Activities (active)
				23	Entertainment and pleasure
				24	Group membership and community ties
		23	**Spontaneous**	17	Activities (active)
				16	Comfort level
		24	**Sustained**	25	Stewardship
				26	Co-operation
				27	Participation and engagement
				38	Space maintenance
4	**Sociability**	25	**Interactive**	28	Interaction with human\nature
		26	**Friendship**	29	Social ties and Friendship
		27	**Pride**	30	Sense of pride
		28	**Neighbourly**	24	Community ties and group membership
		29	**Co-operation**	26	Co-operation
		30	**Leadership**	25	Stewardship
		31	**Diversity**	31	Diversity and variation
				32	Social cohesion

## Methods

### Methodology

**Fig. 2:**
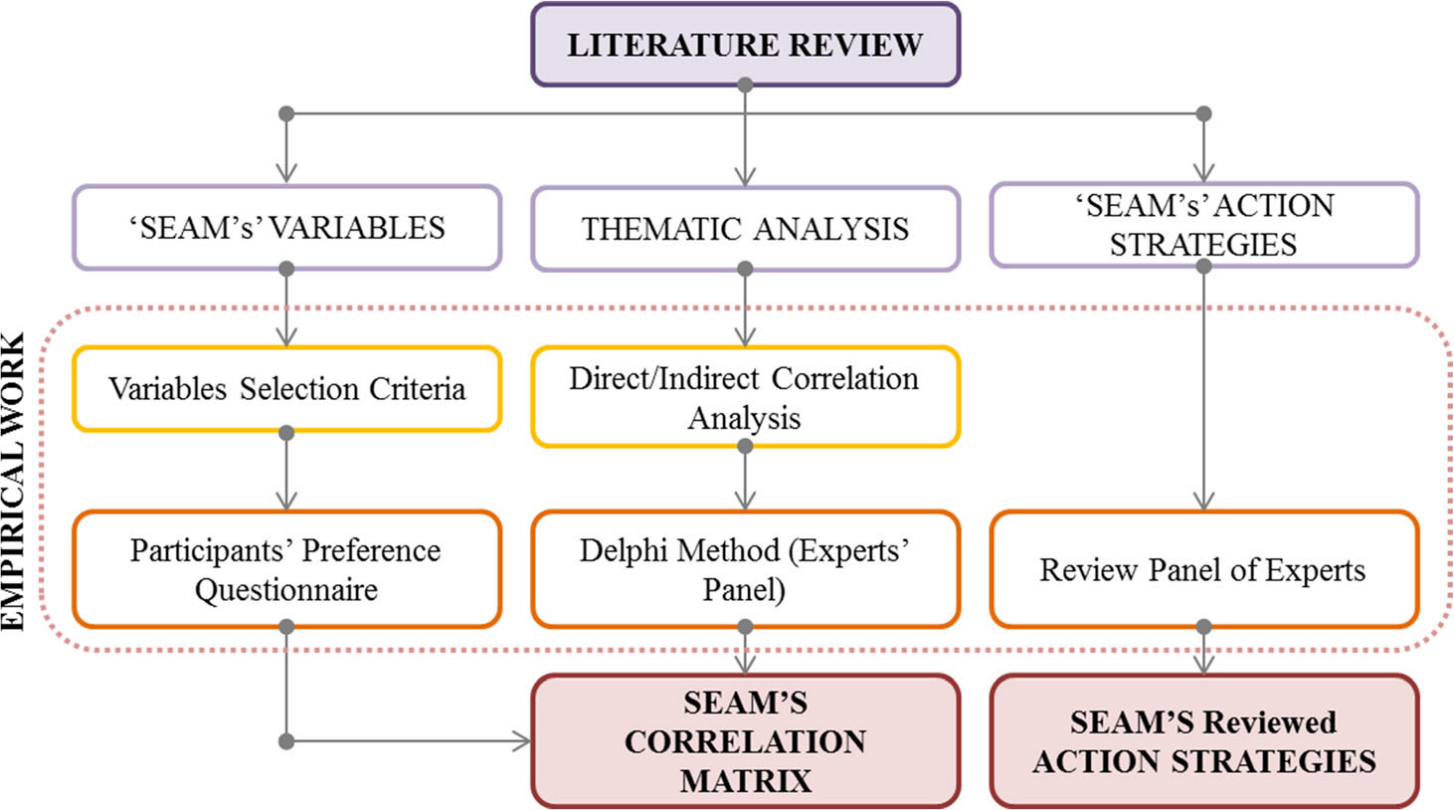
Paper methodology graph

The methodology comprises an in-depth literature search for previous work on ecology and social ecology to scan the field and understand where the Egyptian situation lays within these approaches. In this phase, the paper explored the current approaches and theories of open spaces development. It also went in-depth into scanning for existing design and strategic notions and looked at current examples that could be implemented in the Egyptian context. In order to achieve the main aim, the paper was divided into multi-layered activities [25] (Fig. 2). The first layer is concerned with extracting preliminary themes through readings, descriptions, and significant quotes. Concrete relations were indicated in the texts and tagged with preliminary themes. The second layer is composed of a detailed analysis of the extracted preliminary themes that are coded. The third layer is mainly concerned with finding the emergent common themes and recurrences. Those themes provide interrelations and allow for a holistic understanding of social ecology practice in open spaces [26]. This section starts by explaining quantitative and qualitative approaches such as variables’ synthesis, participants’ questionnaires, and Delphi method, as well as thematic analysis [27].

### Case study selection criteria

**Fig. 3:**
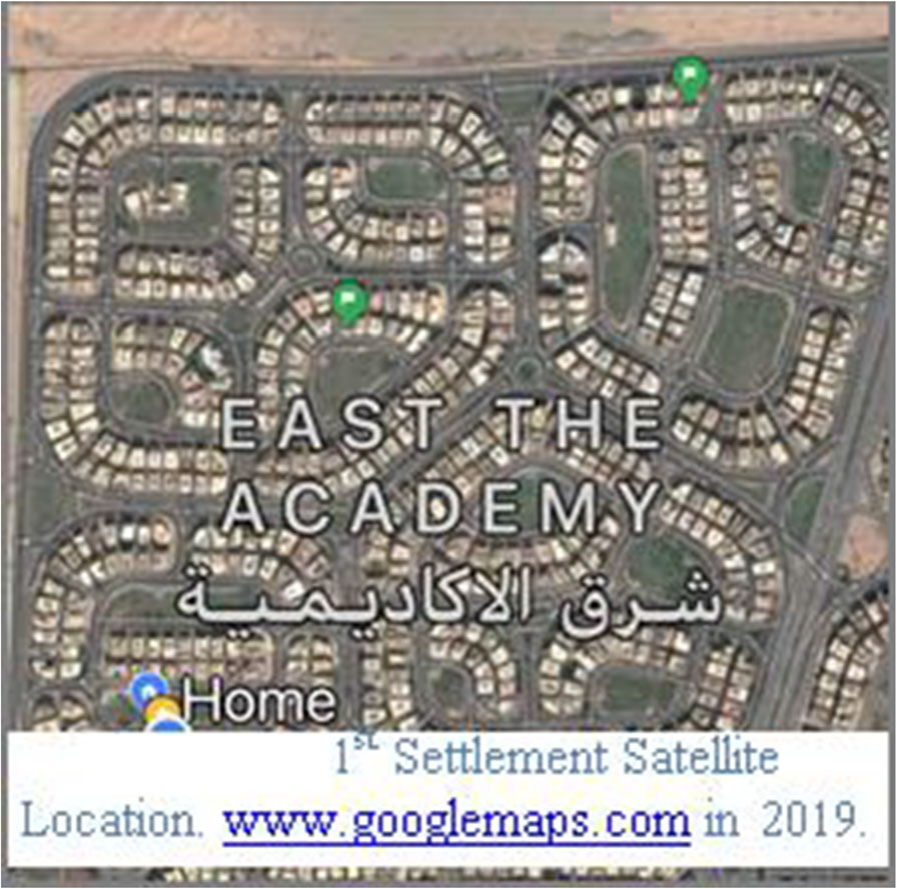
1st settlement satellite location. www.googlemaps.com in 2019

The “East Academy in New-Cairo” was selected as a case study for the empirical work, since it represents an example of a new developing community with a need for understanding to its underlying community needs. East and South Academy are districts within the 1st settlement in New Cairo. These two districts consist of multiple neighbourhoods with concentric urban design. Most of buildings are villas with max height of four floors and at least a front yard. As shown in Fig. 3 [28], these villas are clustered around a shared neighbourhood green space.

## Quantitative and qualitative methods

### S.E.A.M’s variables selection

#### Phase 1 of variables selection (comparative analysis with “PPS”)

A comparative analysis between “PPS” attributes and “S.E.A.M’s” variables are examined to identify the common patterns which represent the latest method of practice concerning open spaces’ design. The outcome of this comparison is presented in Table 6 which manifests a sum of 38 variables common between both “PPS” and “S.E.A.M” methods. These common variables will be processed later using “participants” preference questionnaire’ method to identify 10 variables with max importance and effectiveness from participants’ subjective view.

#### Phase 2 of variables selection (Participants’ Preference Questionnaire)

Preference questionnaire is a quantitative method that is considered a second filtering tool for “S.E.A.M’s” 38 variables. This step targets to reduce the number of traced variables to a sensible amount. The formation of preference questionnaire consists of four main sections.

Section 1 contains gathering of personal data such as age, gender, occupation, education, familiarity with space, participant type (resident, user, or expert), and years of contact with space, to gain a better insight about participant’s characteristics and background. Section 2 contains rating of twelve “Physical and Spatial Elements” (Table 3), such as plantation and vegetation, landmarks and attraction points, enclosure and openness, and space furniture. Section 3 contains rating of twenty “Social, Cultural, and Psychological Aspects” (Table 4), such as safety and security, visibility and surveillance, crime prevention, comfort level, and activities. At last, section 4 contains rating of six “Ecological and Natural Variables” (Table 5), such as green infra-structure, blue infra-structure, and waste management [29].

**Fig. 4:**
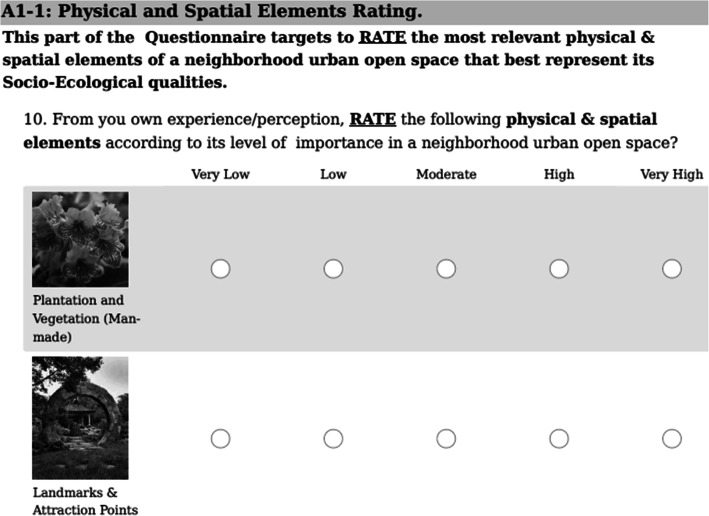
Example of Participants’ Preference Questionnaire

Moreover, the scale that is used through the entire survey is a five point rating scale with numerical representation from (1 to 5) to facilitate results’ quantification. The breakdown of the scale is as follows: very low (= 1), low (= 2), moderate (= 3), high (= 4), and very high (= 5). The previously stated numbers represent variables’ importance according to participants (user, resident, or expert) preference [30]. In addition, a graphical representation of each variable was presented to facilitate the process of recognition and avoid terms’ misperception among all participants (Fig. 4).

### S.E.A.M’s model correlation analysis

#### Phase 1 of correlation themes (thematic analysis)

**Fig. 5:**
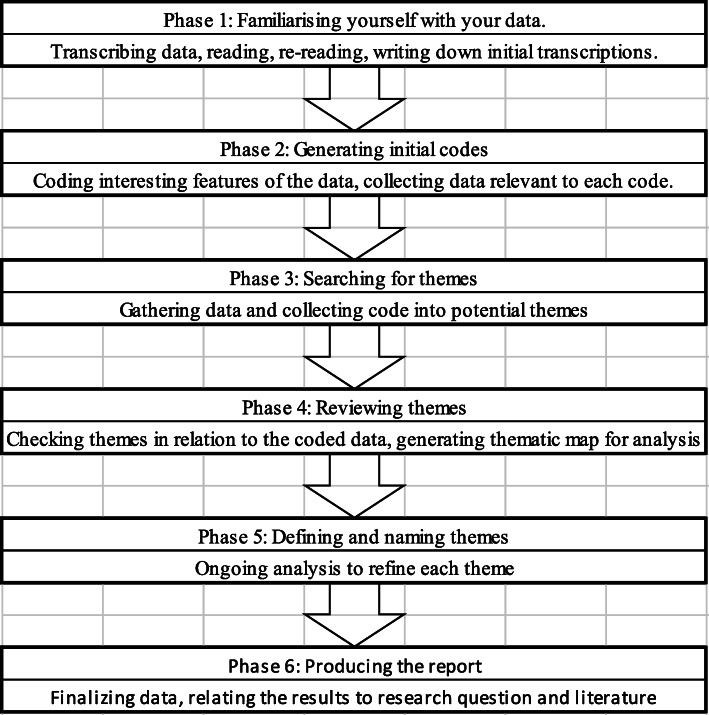
“Phases of thematic analysis by Braun and Clarke in 2008.” To be adopted for the research at hand

Thematic analysis is usually used in qualitative research, according to Braun and Clarke it is defined as “A method for identifying, analysing and reporting patterns within data”. A theme captures what is considered important about the data in relation to the proposed research question and represents some level of patterned meaning within the data set. It minimally organizes and describes data rich in details through its theoretical freedom. “Thematic analysis provides a flexible and useful research tool, which can provide a rich and detailed, yet complex, account of data” [24]. The following diagram represents the adopted sequence of phases (six main phases) for good thematic analysis (Fig. 5).

In this paper, the translation of the previously mentioned steps is as follows: phase one, a cohesive reading and re-reading through literature took place with writing down initial transcriptions that is presented in Table 8 under the title “Description”; phase two, coding data and highlighting features that are related to certain categories are clarified under the name “Interpretation”; and phase three, collecting data into potential themes as presented in Table 8 in the column “Possible themes”. Moreover, in order to validate the previously mentioned “Possible Themes”, a review process took place. This review process was divided into two steps (step four and step five) that were undertaken by the author and again by group of experts (review phase 2, Delphi method). Finally, phase six was conducted to relate the thematic analysis results with the correlation analysis of S.E.A.M’s variables which are presented in Table 9 “Relation Matrix of S.E.A.M’s variables”.

#### Phase 2 of correlation themes (Delphi method)

This paper required expert’s consultation in two rounds of Delphi survey; the first round was to rate the importance of socio-ecological indicators to assess urban open spaces. The second round was to review thematic analysis and provide feedback to validate author’s correlational analysis. A multidisciplinary group of sixty participants and ten experts rated the importance and themes of 38 indicators with response rates of 60% and 75% in the two rounds. Delphi techniques have been used to develop S.E.A.M.’s socio ecological indicators among expert group. A Delphi involves an anonymous survey using questionnaires with controlled feedback to allow rotation within a panel of experts. It is also understood as a tool for reaching expert’s opinion through scientific discourse in complex situations in which the relations between variables are not clearly evident [25]. The Delphi study presented here was developed in a structured format in order to assess a list of pre-defined indicators drawn from the literature.

#### Delphi procedure

Invitation letter was sent to the nominated participants by email to complete the rating process. They were asked to give their demographic information. The participants were to rate the importance of each indicator on a 5-piont scale (1 = very low important to 5 = very high important). The questionnaire included a section where the participants could add free text comments. A reminder email was sent in each round. At the second round, the experts were presented with feedback results for each indicator rated in the first round. Indicators were extracted from the literature reviewed and subjected to consultation about comprehensiveness in a pilot rating exercise from three volunteered experts.

## Results and discussion

### Variables selection criteria results

Results of the “Participants” Preference Questionnaire’ helped in categorizing the socio-ecological attributes into groups according to majority of votes. High and very high scores were represented by 1 in the “majority rating column”, while lower scores were represented by 0. Moreover, another filtering technique was applied following each attribute tendency towards low or high rating score. By calculating the mean values of the attributes; attributes with mean scores lower than 3.5 were considered “moderate with tendency to low”; on the other hand, attributes with mean scores higher than 3.5 and lower than 4.0 were considered “moderate with tendency to high”. These “Means’ Tendencies” were translated to one-digit representation either 1 or 0 in the column “Tendency to High” (Table 7). As a direct result from the previously explained filtering technique, a number of attributes were highlighted (10 attributes in total). These socio-ecological attributes are accessibility, walkability, safety and security, crime prevention, comfort level, sense of beauty, green infra-structure, waste management, pedestrian paths, and space maintenance.

**Table 7:** Participants’ Preference Questionnaire results summary

Reduction method					Rating questionnaire results summary							
Final results	STDV chart	Semi-final result	H\tendency to H	Majority rated H/VH			**Very Low (1)**	**Low (2)**	**Moderate (3)**	**High (4)**	**Very High (5)**	**Means**
2	1	1	1	0	**Physical and spatial elements**							
					PSE01	Plantation and vegetation	5.0%	0.0%	46.0%	27.0%	22.0%	3.6
1	0	1	0	1	PSE02	Landmarks and attraction points	9.0%	7.0%	28.0%	42.0%	14.0%	3.5
0	0	0	0	0	PSE03	Enclosure and openness	2.0%	15.0%	44.0%	26.0%	13.0%	3.3
0	0	0	0	0	PSE04	Historical elements	46.0%	18.0%	31.0%	5.0%	0.0%	2.0
1	0	1	0	1	PSE05	Space furniture	11.0%	10.0%	25.0%	42.0%	12.0%	3.3
3	1	2	1	1	PSE06	Accessibility	0.0%	3.0%	27.0%	43.0%	27.0%	3.9
0	0	0	0	0	PSE07	Continuity	12.0%	11.0%	52.0%	16.0%	9.0%	3.0
0	0	0	0	0	PSE08	Proximity	7.0%	7.0%	47.0%	25.0%	14.0%	3.3
1	0	1	0	1	PSE09	Connectivity and movement	4.0%	14.0%	27.0%	39.0%	16.0%	3.5
1	0	1	0	1	PSE10	Wayfinding and navigation	6.0%	14.0%	22.0%	41.0%	17.0%	3.5
3	1	2	1	1	PSE11	Walkability	9.0%	12.0%	7.0%	39.0%	33.0%	3.8
1	0	1	0	1	PSE12	Mixed uses and services	7.0%	15.0%	24.0%	34.0%	20.0%	3.5
0	0	0	0		**Social and cultural aspects**							
3	1	2	1	1	SCA01	Safety and security	1.0%	9.0%	11.0%	40.0%	39.0%	4.1
1	0	1	1	0	SCA02	Visibility and surveillance	3.0%	0.0%	44.0%	32.0%	21.0%	3.7
3	1	2	1	1	SCA03	Crime prevention	5.0%	9.0%	9.0%	37.0%	40.0%	4.0
3	1	2	1	1	SCA04	Comfort level	2.0%	5.0%	10.0%	47.0%	36.0%	4.1
1	0	1	1	0	SCA05	Activities (active/passive)	2.0%	12.0%	32.0%	31.0%	23.0%	3.6
0	0	0	0	0	SCA06	Motivation	2.0%	17.0%	40.0%	29.0%	12.0%	3.3
0	0	0	0	0	SCA07	Heritage values	12.0%	24.0%	41.0%	16.0%	7.0%	2.8
0	0	0	0	0	SCA08	Memories	7.0%	30.0%	42.0%	14.0%	7.0%	2.8
0	0	0	0	0	SCA09	Space attachment	10.0%	14.0%	48.0%	17.0%	11.0%	3.1
3	1	2	1	1	SCA10	Sense of beauty	3.0%	5.0%	21.0%	55.0%	16.0%	3.8
0	0	0	0	0	SCA11	Entertainment and pleasure	5.0%	2.0%	41.0%	39.0%	13.0%	3.5
0	0	0	0	0	SCA12	Group membership and community ties	8.0%	17.0%	40.0%	29.0%	6.0%	3.1
0	0	0	0	0	SCA13	Stewardship/leadership	20.0%	26.0%	40.0%	9.0%	5.0%	2.5
0	0	0	0	0	SCA14	Co-operation.	8.0%	13.0%	37.0%	31.0%	11.0%	3.2
1	0	1	0	1	SCA15	Participation and engagement	9.0%	15.0%	28.0%	34.0%	14.0%	3.3
2	1	1	0	1	SCA16	Interaction with human/nature	8.0%	10.0%	19.0%	46.0%	17.0%	3.5
1	0	1	0	1	SCA17	Social ties and friendship	7.0%	6.0%	31.0%	38.0%	18.0%	3.5
0	0	0	0	0	SCA18	Sense of pride	3.0%	16.0%	40.0%	28.0%	13.0%	3.3
0	0	0	0	0	SCA19	Diversity and variation	8.0%	12.0%	40.0%	30.0%	10.0%	3.2
0	0	0	0	0	SCA20	Social cohesion	9.0%	24.0%	43.0%	21.0%	15.0%	3.2
0	0	0	0		**Ecological and natural variables**							
3	1	2	1	1	ENV01	Green infra-structure (greenery)	3.0%	3.0%	24.0%	40.0%	30.0%	3.9
1	0	1	0	1	ENV02	Blue infra-structure (water features)	15.0%	14.0%	18.0%	34.0%	19.0%	3.3
3	1	2	1	1	ENV03	Waste management	4.0%	10.0%	14.0%	43.0%	29.0%	3.8
0	0	0	0	0	ENV04	Recycled materials	15.0%	23.0%	24.0%	19.0%	19.0%	3.0
3	1	2	1	1	ENV05	Pedestrian paths	12.0%	2.0%	15.0%	36.0%	35.0%	3.8
3	1	2	1	1	ENV06	Space maintenance	5.0%	6.0%	22.0%	30.0%	37.0%	3.9

### Correlations’ extraction

#### Possible themes as a result of thematic analysis

In order to track possible themes between varieties of socio-ecological attributes, a comprehensive thematic analysis was conducted (Table 8), in addition to creating multiple thematic tables. In this paper, a sequenced of processes for analysing the input data from literature were adapted. Also, specific phrases related to the paper questions were selected and quoted for further investigation. These phrases are displayed under the title “Description” in Table 8. This table is a part of fourteen analytical tables that were finalized by the researcher and reviewed by a team of experts. The “Interpretation” column is a breakdown of what was mentioned in the “Description”. This breakdown can be considered guidance to certain relations/correlations between variables. Moreover, “Possible themes” were extracted with more focus on one to one direct relations. These direct relations were the starting point to search for validation by experts in later steps.

**Table 8:** Thematic analysis and extraction of possible themes

Thematic analysis: physical, social and ecological themes extraction				
**Description source**		**Description**	**Interpretation**	**Possible themes by Delphi**
1	Dunnett, 2002	“some active recreation, such as jogging, may take place in an open space as an individual activity or in small groups, walking may be undertaken by individuals or in familial or friendship groups….organised walking groups…..‘Walking for health’ schemes.”	There is a relation between **the existence of an open space with activities and recreation**; also these **activities** create opportunities for **group interaction.**	*Direct relation between open spaces and active recreation. *Direct relation between activities and group membership. *Indirect relation between open spaces and group membership.
		“These events may be organised by community groups…….These events help to enhance the value that a community attributes to its urban open spaces….. Local authorities do keep records of events and these, however, reveal that many events have a focus for a particular cultural or religious group.....mental restoration or catching up with community news from other adults and children met along the way.”	There is an association between **events organization and the community value for an open space** also these events play **role in cultural exposure, mental restoration, and human interaction.**	*Direct relation between community events and space attachment. *Direct relation between community events and mental restoration, cultural representation, human interaction.
2	Greenhalgh and Worpole, 1995	“Taking children to play is one of the main reasons for visiting urban open spaces for many people…”	Existence of **urban open spaces** such as **urban parks and playing fields** is associated with **physical activities** such as children’s play.	*Direct relation between green infra-structure and physical activities.
3	Research on children by Taylor, 1998	“Outdoor play is shown to be important for social development including collaborative skills, negotiating skills, confrontation and resolution of emotional crises, management of conflicts and development of moral understanding….important for the development of cognitive skills such as language and language comprehension, experimentation and problem solving techniques.”	**Physical activities** are associated with children’s **social development** such as **collaboration, negotiation, confrontation, psychological health, moral understanding and management of conflicts.** Also associated with **cognitive skills** development, **comprehension and experimentation.**	*Direct relation between physical activities and interaction, Co-Operation, social ties, comfort level, engagement, values and norms.
4	Noschis, 1992	“Considered to be a significant aspect of play as a means of bringing children closer to the adult world and helping children to construct their own identity.”	**Outdoor activities** construct **personal identity** as well as **encouraging personal character, integration and interaction with adults.**	*Direct relation between physical activities and personal identity, interaction and group membership.
5	The National Playing Fields Association (NPFA), 2000	“assert the importance of play in the outdoor environment in providing opportunities for freedom, large-scale physical activities and different challenges …”	Existence of **urban open spaces** allows **feeling of freedom**.	*Direct relation between green infra-structure and personal identity and (active\passive) activities.
6	Opie and Opie, 1969	“On top of this is the experience of starting a game—gathering people to join in—which can in itself become a game”	**Group activities** encourage **feeling of membership** while **creating interaction and engagement opportunities**.	*Direct relation between activities and group membership, interaction and engagement.
7	Hart, 1979	“Hart investigated four areas of interaction with the environment: spatial activity; place knowledge; place values and feelings and place use. Underlying this research was a fundamental belief that children experience the landscape in a very personal way.”	Children experience landscape in a personal way using **activities to enhance their space knowledge, values and feelings** toward the occupied space.	*Direct relation between green infra-structure and activities, place attachment, values and norms, personal identity, memories and space image.
8		“Relationships with the children were further developed when Hart joined the children in the exploration of their local environment, to the extent that when interviewing Hart was treated as part of the children’s ‘gang’.”	Sharing outdoor **activities enhances group social ties and the feeling of belonging.**	*Direct relation between interaction and group membership, social ties friendship, comfort level and sense of safety.

#### Correlation matrix and Delphi method results

A “Correlation Matrix” is a graphical representation of concluded themes between S.E.A.M’s variables; these themes were reviewed and validated by Delphi method. The review of these themes was conducted by a group of ten experts. Those experts provided researchers with a written feedback in two formats. The first format was either confirming or denying the correlation provided from thematic analysis. The second format was composed of added notes for other possible themes or correction for the extracted written one.

After collecting all experts’ feedbacks; a final confirmation round was published among the same experts’ panel to share a final insight. The confirmation round was conducted by email to facilitate sharing all comments among the panel. At last, these correlations are represented in Tables 9, 10, 11, and 12 as a result from both thematic analysis and Delphi method. These correlations are represented with two symbols either (**√**) as a mean for proofed correlation or (**X**) as a mean for possible correlation.

**Table 9:** Proofed and possible correlations symbol and meaning key

Symbol	Meaning
**√**	Proofed correlation by thematic analysis and questionnaire.
**X**	Possible correlation either by thematic or questionnaire.

**Table 10:** Correlation matrix between the chosen ten S.E.A.M’s variables and the 38 variables of PPS-S.E.A.M’s attributes

Urban physical elements	Landmarks and attraction points	Boundaries (enclosure and openness)	Historical elements	Plantation and vegetation	Space\street furniture	Mixed uses\services	Navigation, way finding, and recognition.	Accessibility	Walkability	Proximity	Continuity	Connectivity and Movement
Accessibility	x	x		x			x		x			x
Walkability	x	x		x			x	x		x		x
Sense of beauty			**√**	**√**								
Safety and security		**√**		**√**			**√**	**√**		**√**		**√**
Comfort Level			**√**	**√**	x	x		**√**	**√**	x		x
Crime prevention		**√**					**√**	**√**	**√**	**√**		**√**
Garden\parks	x	x		**√**			x	**√**	**√**	x		x
Space maintenance					x			x	x			x
Waste management				x				x	x			x
Pedestrian networks	x	x		x	x	x	x	x	**√**	x	x	x

**Table 11:** Correlation matrix between the chosen ten S.E.A.M’s variables and the 38 variables of PPS-S.E.A.M’s attributes

Social and cultural aspects	Heritage values	Memories	Sense of beauty	Safety and security	Sense of pride	Interaction (with human\nature)	Comfort level	Activities (active and passive)	Co-operation	Motivation	Social ties and friendship	Place attachment	Entertainment and pleasure	Diversity and variation	Stewardship	Participation and engagement	Social cohesion	Group membership	Crime prevention	Visibility and surveillance
Accessibility							**√**													**x**
Walkability		x	x	x		x	**√**	x		x	x		x					x	x	x
Sense of Beauty	**√**	**√**																		
Safety and security						**√**		**√**			**√**			**√**		**√**	**√**	**√**	**√**	**√**
Comfort level	**√**	**√**	x	x		**√**					x		x			x	x	x	x	
Crime prevention			**√**	**√**		**√**	x	**√**	x		**√**	x		x	x	x	x	x		**√**
Garden\parks	**√**	**√**	x	**√**	**√**	**√**	**√**	**√**	x	x	**√**	**√**	**√**			**√**	**√**	**√**	**√**	x
Space maintenance		x	x	**√**	x	x	x	x				x	x				x	x	**√**	x
Waste management		x	x	**√**	x		x					x	x						**√**	
Pedestrian networks			**√**	x		x	x	x					x							x

**Table 12:** Correlation matrix between the chosen ten S.E.A.M’s variables and the 38 variables of PPS-S.E.A.M’s attributes

Ecological and natural variables	Garden\parks	Urban agriculture	Vertical landscape	Playgrounds\playfields	Incidental spaces	Street landscape	Allotments	Roof-scape\green Roof	Green belt	Natural habitat	Recycled materials	Space maintenance	Waste management	Underground\rain water	Sea, river, lake, waterfalls	Man-maid water sources	Eco-mobility	Pedestrian networks
Accessibility	**√**					x						**x**						x
Walkability	**√**			x		x	x			x		x	x		x	x		**√**
Sense of Beauty																		**√**
Safety and security	**√**				**√**							**√**	**√**				**√**	
Comfort level	**√**	**√**	x	**√**	x	**√**	**√**	x		**√**					x	x		x
Crime prevention	**√**			**√**	x							**√**	**√**		**√**		x	
Garden\parks		x		x	x		x	x			x	x	x	x	x	x		**√**
Space maintenance													x					
Waste management											x	x		x				
Pedestrian networks	**√**					x						x	x		x	x	x	

To conclude, the main outcomes of this paper are proofed and possible correlations between multiple socio-ecological attributes in New Cairo, Egypt. These proofed correlations are extracted from a triangulation method where literature, participants’, and experts’ preferences were counted. It was established that from urban and spatial elements’ correlations, (1) accessibility is correlated with comfort level and gardens, and (2) walkability with comfort level, gardens, and pedestrian network. From social and cultural aspects’ correlations, (1) sense of beauty is correlated with historical values, plants, heritages, memories, and pedestrian network; (2) safety and security are correlated with boundaries, plants, navigation, accessibility, proximity, connectivity, interaction, activities, social ties, diversity, participation, social cohesion, group membership, crime prevention, and visibility; (3) comfort level with historical value, plants, accessibility, walkability, heritage, memories, interaction, gardens, agriculture, playgrounds, street landscape, allotments, and natural habitat; and (4) crime prevention with boundaries, navigation, accessibility, walkability, proximity, connectivity, sense of beauty, safety and security, interaction, activities, social ties, visibility, gardens, playgrounds, space maintenance, waste management, and water features. From ecological and natural variables’ correlations, (1) gardens and parks are correlated with plants, accessibility, walkability, heritage, memories, safety and security, sense of pride, interaction, comfort level, activities, social ties, place attachment, entertainment and pleasure, participation, social cohesion, group membership, crime prevention, and pedestrian network; (2) space maintenance correlated with safety and security, and crime prevention; (3) waste management is correlated with safety and security and crime prevention; and (4) pedestrian network with walkability, sense of beauty, and gardens.

## Conclusions

This paper was set out in order to explore the key preferences of socio-ecological attributes that can potentially contribute in creating a positive impact in urban open spaces of New Cairo, Egypt. The paper has also sought to define social and ecological concepts in urban settings, also to understand the urban physical elements, social and cultural aspects, ecological and natural variables, and their correlations on open spaces of New Cairo’s context using a single case study (East Academy District). Data were collected through a triangulation method using thematic analysis, users’ survey, and experts’ panel. Findings indicate that East-Academy’s open spaces have strong correlations with multiple socio-ecological attributes and that ten urban qualities also showed highest positive measures.

Moreover, this paper laid hands on hidden correlations between socio-ecological variables. This may lead in the future research to interventional action strategies for residential open spaces in which both the variable itself and its strongest relation are taken into account. This is achieved by conducting the thematic analysis method by scanning the literature and highlighting the main extracted themes between the different variables of the socio-ecological ecology variables. Due to the wide variety of socio-ecological variables, a selection technique was applied to nominate the most important ten variables according to participant’s preference. Later, a “correlation matrix” was generated to illustrate the validated correlations of these ten variables: accessibility, walkability, safety and security, crime prevention, comfort level, sense of beauty, green infra-structure, waste management, pedestrian paths, and space maintenance; also, it was validated by panel of experts following Delphi method.

In conclusion, a few limitations of this paper are highlighted to provide a better opportunity for future research. Only one case was investigated; in future research, more than one district could be analysed from different locations across New Cairo and Egypt to see if there are similar results and patterns. This would also help in generalization and in increasing the validity of the results. In addition, a comparison could also be made with districts in different countries to identify possible commonalities between the perceptions of users among different cultures. Furthermore, another objective could be achieved by creating “S.E.A.M’s Action Strategies” from multiple disciplines, urban open spaces, physical and human, social and cultural, natural and environmental, and regenerative design. This “S.E.A.M’s Action Strategies” is open for future research to relate it to “S.E.A.M’s Correlation Matrix” which can be an attempt to conclude design recommendations on how to reach ecological, social, and regenerative enriched open spaces.

## Data Availability

The datasets used and/or analysed during the current paper are available from the corresponding author on reasonable request.

## Abbreviations

PPS: Project of public spaces
S.E.A.M: Socio-ecological action model
COVID-19: Coronavirus disease 2019
PMM: Place making model

## References

[1] Janlin L (2012) The urban sociology reader. Routledge, Millton Park, Oxfordshire

[2] Bronfenbrenner U (1993) Ecological models of human development. International Encyclopedia of Education:37–43

[3] Ugolini F (2020). Effects of the COVID-19 pandemic on the use and perceptions of urban green space: an international exploratory study. Urban Forestry and Urban Greening.

[4] Geary RS (2021). A call to action: improving urban green spaces to reduce health inequalities exacerbated by COVID-19. Prev Med.

[5] Popenoe G a (1970). Neighborhood, city and metropolis: an integrated reased in urban sociology.

[6] Clark J (2000) A social ecology. Environmental Philosophy, Englewood Cliffs

[7] Thomson (2000) Ecology, community and delight, London

[8] Duncan OD (1961). From social system to eco system.

[9] Carmona M (2021). Public places urban spaces- the dimensions of urban design.

[10] Bakker I (2020). Pleasure, arousal, dominance: Mehrabian and Russell revisited. Curr Psychol.

[11] Herzog TR (1992). A cognitive analysis of preference for urban spaces. J Environ Psychol.

[12] D'Acci S, L. (2021). Preferring or needing cities? (Evolutionary) psychology, utility and life satisfaction of urban living. City Cult Soc.

[13] Lynch K (1960). The image of the city.

[14] Koseoglu E (2011). Subjective and objective dimensions of spatial legibility. Procedia Soc Behav Sci.

[15] Kopec D (2012) Environmental psychology for design. Fair Child Publications, New York City

[16] Ellery PJ (2020). Toward a theoretical understanding of placemaking. International Journal of Community Well-Being.

[17] Spaces, P. f. (2019, 1 10). What makes a successful place? Retrieved from pps.org: http//www.pps.org/article/grplacefeat

[18] Santayana G (1955). The sense of beauty being the outline of aesthetic theory.

[19] Alexander C (1977). A pattern language towns, buildings, construction.

[20] Rapoport A (2016). Human aspects of urban form.

[21] Newman O (1996) Defensible space crime prevention through urban design. MacMillan Publishing Company, London

[22] Yeang K (2009). Ecological master planning.

[23] Mostafavi M (2016). Ecological urbanism.

[24] Woolley H (2013). Urban open spaces.

[25] Timma L (2015). Combined and mixed methods research in environmental engineering: when two is better than one. Energy Procedia.

[26] Clarke VB (2008) Using thematic analysis in psychology. Qual Res Psychol:77–101

[27] Yacob MR (2015) Delphi method of developing environmental well-being indicators. Procedia Environ Sci 30

[28] 1st settlement satellite location. (2019, 8 4). Retrieved from Google Maps: www.googlemaps.com.

[29] Creswell J (2002). Research design: qualitative, quantitative, and mixed methods.

[30] Scholz R (2002) Embedded case study methods integrating quantitative and qualitative knowledge, London

